# Dysregulated zinc homeostasis and microadenomas in the anterior pituitary: pathological insights into suicide risk

**DOI:** 10.3389/fpsyt.2024.1446255

**Published:** 2024-08-13

**Authors:** Hiram Tendilla-Beltrán, Patricia Aguilar-Alonso, Carlos Alejandro Hernández-González, Eduardo Baltazar-Gaytán, Ana A. Orduña, Humberto Nicolini, Fernando García-Dolores, Gonzalo Flores

**Affiliations:** ^1^ Instituto de Fisiología, Benemérita Universidad Autónoma de Puebla (BUAP), Puebla, Mexico; ^2^ Facultad de Ciencias Químicas, Benemérita Universidad Autónoma de Puebla (BUAP), Puebla, Mexico; ^3^ Hospital Regional “1° de Octubre”, Instituto de Seguridad y Servicios Sociales de los Trabajadores del Estado (ISSSTE), Mexico City, Mexico; ^4^ Facultad de Medicina, Universidad Veracruzana (UV) Región Córdoba – Orizaba, Campus Ciudad Mendoza, Mendoza, Veracruz, Mexico; ^5^ Escuela Superior de Medicina, Centro de Estudios Tecnológicos y Universitarios del Golfo, Orizaba, Veracruz, Mexico; ^6^ Instituto Nacional de Medicina Genómica (INMEGEN), Mexico City, Mexico; ^7^ Instituto de Ciencias Forenses (INCIFO), Tribunal Superior de Justicia de la Ciudad de México (TSJCDMX), Mexico City, Mexico

**Keywords:** suicide, zinquin, dithizone, adenoma, HPA-axis

## Abstract

**Background:**

Suicide is a significant public health problem influenced by various risk factors, including dysregulation of the hypothalamus-pituitary-adrenal (HPA) axis. Zinc (Zn), essential for pituitary function in hormone synthesis and release, has been linked to suicide, with studies noting reduced serum levels and altered brain transport mechanisms. Despite Zn’s crucial role in pituitary function and its involvement in suicidal behavior, information on pituitary Zn in suicide is scarce. Tumor cells modify Zn dynamics in tissues, and a previous report suggests microadenomas in the anterior pituitary as a risk factor for suicide.

**Methods:**

Histopathological analysis with hematoxylin-eosin stain and histochemical techniques to assess Zn homeostasis were carried out on anterior pituitary postmortem samples from 14 suicide completers and 9 non-suicidal cases.

**Results:**

Pituitary microadenomas were identified in 35% of suicide cases and none in the non-suicidal cases. Furthermore, compartmentalized Zn (detected via dithizone reactivity), but not free Zn levels (detected via zinquin reactivity), was lower in the suicide cases compared to the non-suicidal group.

**Conclusion:**

This is the first report of a potential association between disrupted Zn homeostasis and microadenomas in the anterior pituitary as a feature in suicide and provides critical insights for future neuroendocrine Zn-related research.

## Introduction

Suicide is a public health problem underpinned by a myriad of genetic, biological, sociodemographic, and economic risk factors (1). Among the biological factors, impairments in the hypothalamus-pituitary-adrenal (HPA) axis are some of the most documented. The HPA axis is crucial for stress regulation, a fundamental process necessary for survival (2). However, when stress becomes chronic, it can have deleterious effects on the organism, compromising executive function. Thus, HPA dysfunction is considered a major risk factor for suicide (3).

Among the components of the HPA axis, the pituitary gland arises as the “master gland” due to its capacity to synthesize multiple hormones. This gland is anatomically and functionally characterized by its two lobes: the anterior pituitary (adenohypophysis) and the posterior pituitary (neurohypophysis) (4). The anterior pituitary contains corticotropes, specialized group of cells sensitive to corticotropin-releasing hormone (CRH), which is produced in the paraventricular hypothalamic nucleus. CRH triggers the synthesis of adrenocorticotropic hormone (ACTH), which ultimately leads to the synthesis and release of cortisol and catecholamines in the adrenal glands (5). The synthesis and release of HPA-axis hormones are influenced by various factors, including environmental stress (6) and physiological factors such as age (7) and sex (8). Moreover, the bioavailability of trace elements such as copper (Cu), iron (Fe), manganese (Mn), and zinc (Zn) is crucial for hormone synthesis in the anterior pituitary (9, 10).

Zn, the most abundant trace element in mammalian cells (11), is crucial for various physiological processes, including the function of the pituitary gland. Zn plays a key role in synthesizing, storing, and releasing hormones in the pituitary gland. As a cofactor for enzymes, it is essential for the proper function of the endocrine system. Specifically, Zn regulates the synthesis and secretion of growth hormone, which is vital for body tissue growth and maintenance. Additionally, Zn regulates the synthesis of growth hormone-releasing hormone (GHRH) and thyroid-stimulating hormone (TSH) in the anterior pituitary, which consequently produce growth hormone (GH) and thyroid hormone, respectively (12, 13). Interestingly, lower serum Zn levels have been detected in patients diagnosed with depression (14) and in those with suicidal ideation (15). Additionally, in patients undergoing conventional antidepressant pharmacotherapy (tricyclic antidepressants and selective serotonin reuptake inhibitors), Zn supplementation enhances depressive symptoms (16). These findings, along with the biological role of Zn, suggest that a deficiency in this trace element may contribute to the pathophysiology of depression (17), which is a major risk factor for suicidal behavior, attempts, and completion (1).

Despite the crucial role of Zn in pituitary function and its involvement in the pathophysiology of depression and suicide through the HPA axis, information about the relationship between Zn and suicide remains scarce. A previous report of our group studied Zn levels in pituitary homogenates of suicide completers and no differences were detected in comparison with a control group, neither in anterior nor posterior lobes (18). Additionally, Zn is known to play a role in neurotransmission and synaptic plasticity (19), as well as in anti-inflammatory and antioxidant responses (20, 21) which are critical for brain function and mental health (22–26). Abnormal Zn levels could affect these processes, leading to dysregulation in brain regions associated with suicidal behavior such as the hippocampus (27) and the prefrontal cortex (PFC) (28). However, it is important to study Zn beyond their concentration since the dynamics of this trace element are essential to fulfill its biological function.

In the cells, Zn can be whether in a free or compartmentalized form. This process is regulated by transporters from two families: ZRT- and IRT-like proteins (ZIP or SLC39) and Zn transporters (ZnT or SLC30). These transmembrane proteins mobilize Zn either from the extracellular space into the cell or from the cytosol to intracellular compartments (mainly through ZIP activity), or vice versa (through ZnT activity) (11, 29). In postmortem brain samples of suicide completers, increased protein levels of multiple ZnT isoforms have been reported in the PFC (28). Also, Zn dynamics can be regulated by metallothioneins (MTs), a group of low molecular weight, cysteine-rich proteins that bind Zn and other metals with high affinity, thereby serving as another intracellular reservoir of Zn in cells (30). Interestingly, no differences in MTs levels in the anterior pituitary homogenates of suicide completers compared to a control group were detected (18).

Information on Zn dynamics in the pituitary gland in relation to suicide is not only scarce but also appears inconclusive. However, in 2007, Furgal-Borzych and colleagues reported an increased incidence of anterior pituitary microadenomas in suicide completers, suggesting this condition as a risk factor suicide (31). Despite adenomas being relatively common in the general population (approximately present in 10% of it), with more than 90% of these being microadenomas (less than 10 mm in diameter) (32), this finding is significant because tumorous cells are known to have impaired Zn dynamics (29). Zn plays a fundamental role in cellular signaling and gene expression regulation in the majority of cells (33). Zn directly influences tumor cell proliferation by regulating their gene expression and cell survival, processes that can be influenced by changes in ZnT expression (34). Interestingly, in a previous study from our group, anterior pituitary hyperplasia was observed in young-aged suicide completers using stereological methods (18). However, no histopathological analyses of the samples were conducted to analyze the presence of abnormal structures in the anterior pituitary, including adenomas.

Thus, in this study we conducted a histopathological examination focused on evaluating the integrity of the anterior pituitary, accompanied by histochemical analysis to assess Zn homeostasis in suicide completers, compared to non-suicidal cases from Mexico City.

## Materials and methods

### Post-mortem samples

**Figure 1:**
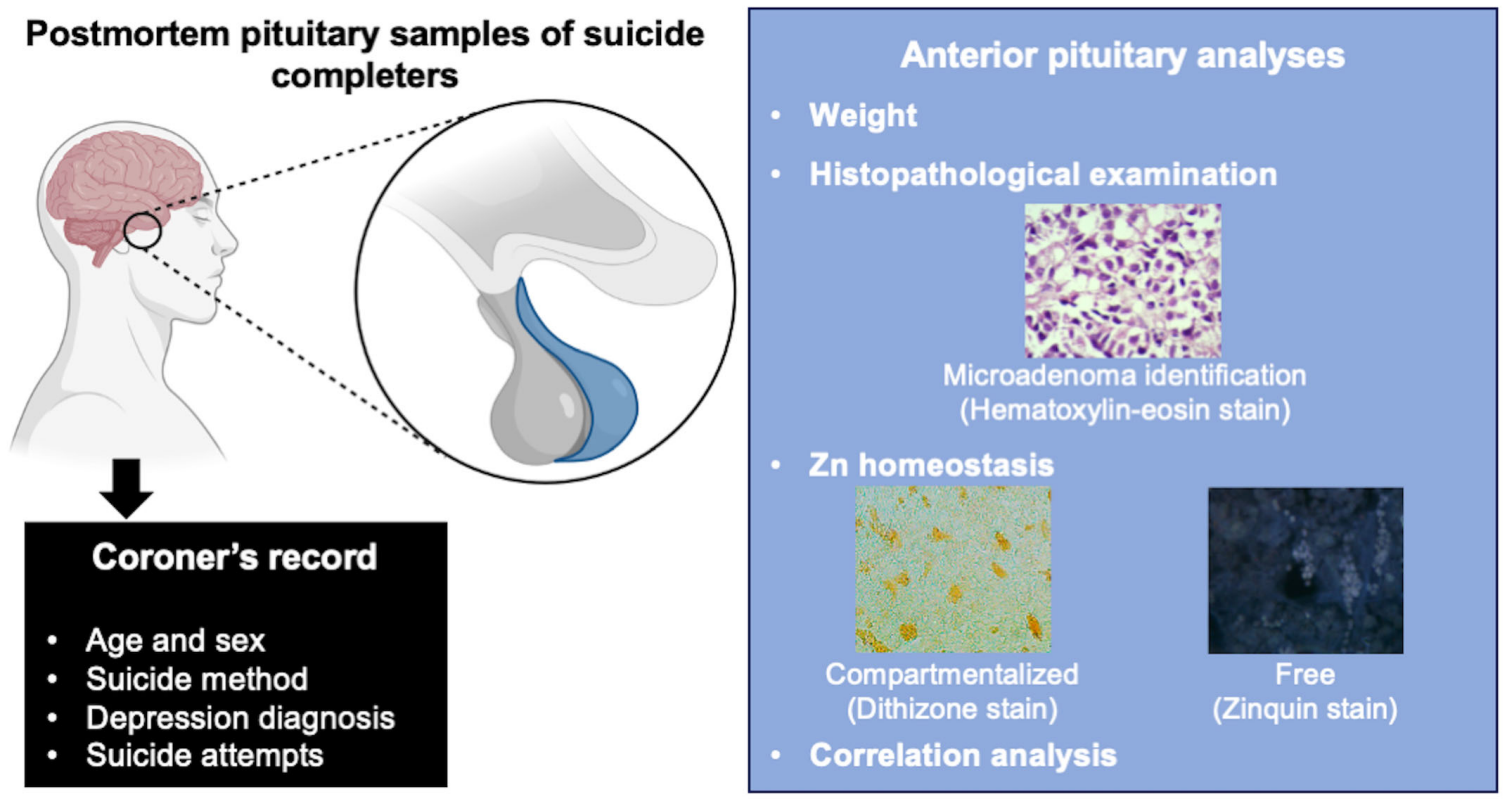
Study design. Pituitary postmortem samples were obtained from 14 suicide completers and 9 non-suicidal cases. The anterior and posterior lobes were dissected and weighed during autopsy. Immediately after dissection, the tissue was fixed in 10% formalin. The anterior pituitary was selected for this study, and histopathological examination was carried out through hematoxylin-eosin-stained coronal sections to analyze the presence of microadenomas. Zn homeostasis was evaluated through dithizone and zinquin stains, which allow the exploration of compartmentalized and free Zn, respectively. Finally, correlation analysis was carried out between continuous variables (age, pituitary weight, zinquin, and dithizone levels) and categorical variables (depression diagnosis, suicide attempts, and adenoma). Coroner’s records for each case included information on age, sex, psychiatric diagnosis, and suicide attempts. The drawings for this figure were created with BioRender.com.

The study design was developed based on previous reports from our group involving postmortem brain samples from suicide completers (18, 35). Pituitary tissue was obtained from 23 cases, including 14 suicide completers and 9 non-suicidal cases, with population characteristics provided in **Table 1**. The tissue was collected during autopsy at the Institute of Forensic Sciences (INCIFO) in Mexico City within 24 hours after death and dissected by a coroner to separate the anterior and posterior lobes. Immediately after dissection, the tissue was weighted and fixed in 10% formalin (pH 7.4) for at least two weeks. All the suicide cases included in this study had a coroner’s record indicating that the cause of death was intentional self-harm by hanging, strangulation, or suffocation (X70 code of the International Classification of Diseases, 10th revision, ICD-10). The coroner’s records contained the death certificate, reports from stressful life situations, toxicology and autopsy reports, police investigation records, and medical reports. This information was gathered from the victims’ families and witness testimonies. To maintain confidentiality, each case was assigned a unique code, and personal data remained inaccessible throughout the study. These records provided information on depression diagnoses (F32 and F33 codes, ICD-10) and suicide attempts (T14.9 code, ICD-10). All outlined procedures received approval from the Research Ethics Committee of the ‘Instituto de Ciencias Forenses del TSJCDMX’ (Conbioética-09-CEI-022-20160823), following national guidelines for health research in humans (NOM-012-SSA3-2012) and the Helsinki Declaration of 1975. Additionally, all data were analyzed anonymously, adhering to ethical standards in biomedical research. Experimental design is shown in **Figure 1**.

**Table 1:** Suicide and non-suicidal cases included in the study.

Suicide completers				
Case	Cause of death	Age	Depression	Suicide attempt
1	Asphyxia by hanging	20-25	No	No
2	Asphyxia by hanging	15-20	Yes	Yes
3	Asphyxia by hanging	15-20	Yes	Yes
4	Asphyxia by hanging	25-30	Yes	Yes
5	Asphyxia by hanging	15-20	No	No
6	Asphyxia by hanging	20-25	No	No
7	Asphyxia by hanging	10-15	Yes	No
8	Asphyxia by hanging	55-60	Yes	Yes
9	Traumatic brain injury*	40-45	No	No
10	Traumatic brain injury*	45-50	No	No
11	Asphyxia by hanging	15-20	Yes	Yes
12	Asphyxia by hanging	25-30	Yes	No
13	Asphyxia by hanging	40-45	No	No
14	Asphyxia by hanging	35-40	No	No
Non-suicidal				
1	General visceral congestion	15-20	No	No
2	Pulmonary thromboembolism	40-45	No	No
3	Carbon monoxide poisoning	15-20	No	No
4	Cerebrovascular accident	10-15	No	No
5	Conjunction of trauma**	25-30	No	No
6	General visceral congestion	50-55	No	No
7	Cerebrovascular accident	45-50	No	No
8	Conjunction of trauma**	30-35	No	No

*Because of jumping from a considerable height. **Because of a traffic accident.

### Hematoxylin-eosin stain

After embedding the formalin-fixed tissue in paraffin, 7 μm coronal sections of the anterior and posterior pituitary were obtained using a microtome (5040, Bright Instrument Company, UK) and placed on glass slides previously coated with gelatin (Sigma-Aldrich, P8920). For all stains described in this study, a rehydration protocol was performed as follows: xylene, ethanol-xylene (50:50), and decreasing ethanol concentrations (absolute to 70%) followed by a final rinse in distilled water. For the hematoxylin-eosin stain, hydrated sections were washed twice with PBS (0.1 M) for 5 minutes during each wash, then stained as follows: immersion in hematoxylin for 1 minute, followed by a rinse in distilled water, and subsequent immersion in 1% acid ethanol (1 ml of 37% HCl per each 99 ml of 96% ethanol) three times. After being washed with distilled water, the samples were dipped in 1% sodium bicarbonate (6 washes until a blue color appeared on the samples), followed by immersion in eosin for 2 minutes. Subsequently, samples were dehydrated using 96% and absolute ethanol, ethanol-xylene, and xylene. Finally, they were mounted with synthetic resin for microscopy (18).

### Zinquin

Freshly prepared zinquin ethyl ester (5 mM in DMSO, Sigma-Aldrich, Z2251) was diluted in PBS (0.1 M) to a final concentration of 25 μM. This solution was directly applied to the slides until they were fully covered for 30 minutes. After removing the zinquin solution, samples were washed three times with PBS (0.1 M) and mounted with VECTASHIELD^®^ (Vector Laboratories, H-1000-10). A fluorescence microscope was used to detect zinquin at excitation wavelengths of 351-358 nm and emission at 460 nm (36).

### Dithizone

Dithizone staining was conducted as follows: 1 mg of dithizone (Sigma-Aldrich, 43820) was dissolved in 1 ml of DMSO and then diluted (1:10) in PBS (0.1 M) to create the working solution. Samples were incubated with the dithizone solution for 50 minutes at 37 °C, rinsed with deionized water, mounted, and analyzed using an optical microscope (36).

### Image analysis

Hematoxylin-eosin-stained sections were analyzed by a forensic physician to identify any histopathological characteristics present in the samples. Dithizone and zinquin-stained sections were analyzed by a trained observer who was blinded to the experimental conditions. In all cases, analyses were performed on six consecutive slices from a 1-in-10 series taken from the anterior (anteroposterior from 0-2.6 mm), middle (anteroposterior from 2.6-5.2 mm), and posterior (anteroposterior >5.2 mm) regions. Dithizone and zinquin images were processed using Image J (NIH) to determine optical density levels per field after adjusting the threshold in 8-bit images. A total of 12 fields were analyzed per slice. These procedures were conducted following stereological methods for human pituitary samples as previously reported (18).

### Statistical analyses

Suicide characteristics and clinical information were reported using frequencies and percentages. Zn reactivity is presented as mean arbitrary units (A.U.) ± the standard error of the mean (SEM). A D’Agostino & Pearson test was employed to verify the normal distribution of the data. Fisher’s exact test was utilized to determine adenoma prevalence, depression etiology, and suicide attempt in the suicide condition, while Student’s t-test was employed for assessing weight and Zn reactivity comparisons. Additionally, Pearson’s correlations between continuous variables (age, pituitary weight, zinquin and dithizone levels) were analyzed for control cases, and Spearman correlations between continuous and categorical variables (depression diagnosis, suicide attempts, and adenoma; presence was considered 1 and absence 0) were analyzed for suicide cases. Due to the lack of female cases, the sex variable was excluded for Spearman correlations. Effect sizes were computed using Cohen’s d test (d), with values of d > 0.8 indicating a substantial effect size (37). A p-value < 0.05 was set as the statistical threshold.

## Results

### Characteristics of the included suicide and non-suicidal cases

The characteristics of the samples indicate that, among suicide completers, the most common method of death was asphyxia by hanging (85.7%), followed by traumatic brain injury (14.3%). Regarding gender, 92.8% were male, and 7.2% were female. Concerning age, 35.6% of the samples were under 18 years old, with an equal proportion over 30 years old, and 28.6% in the 18-30 age group. The mean age for these samples was 29.42 years (SD = 14.03), ranging from 13 to 56 years. For the non-suicidal group, the causes of death were varied: 33.3% resulted from a conjunction of trauma, 22.2% from general visceral congestion, and an equal percentage from cerebral hemorrhage. Additionally, 11.1% were attributed to pulmonary thromboembolism and carbon monoxide poisoning each. Similar to the samples of completed suicides, the male group predominated (66.6%) compared to the female group (33.3%). Lastly, in terms of age, the highest percentage of cases was in the 18-30 age group with 44.4%, followed by the >30-year-old group with 33.3%, and the <18 years old group with 22.2%. The mean age for the non-suicidal cases was 30.38 years (SD = 14.23), ranging from 13 to 50 years. Additionally, 50% of suicide cases were related to depression as the etiology (Fisher’s exact test, two-tailed, p = 0.0225), and 5 suicide cases (35%) had a history of at least one previous suicide attempt. However, these two variables did not show a statistically significant correlation with one another (Fisher’s exact test, two-tailed, p = 0.1154).

### Increased weight and microadenomas were detected in the anterior pituitary in suicide cases

**Figure 2:**
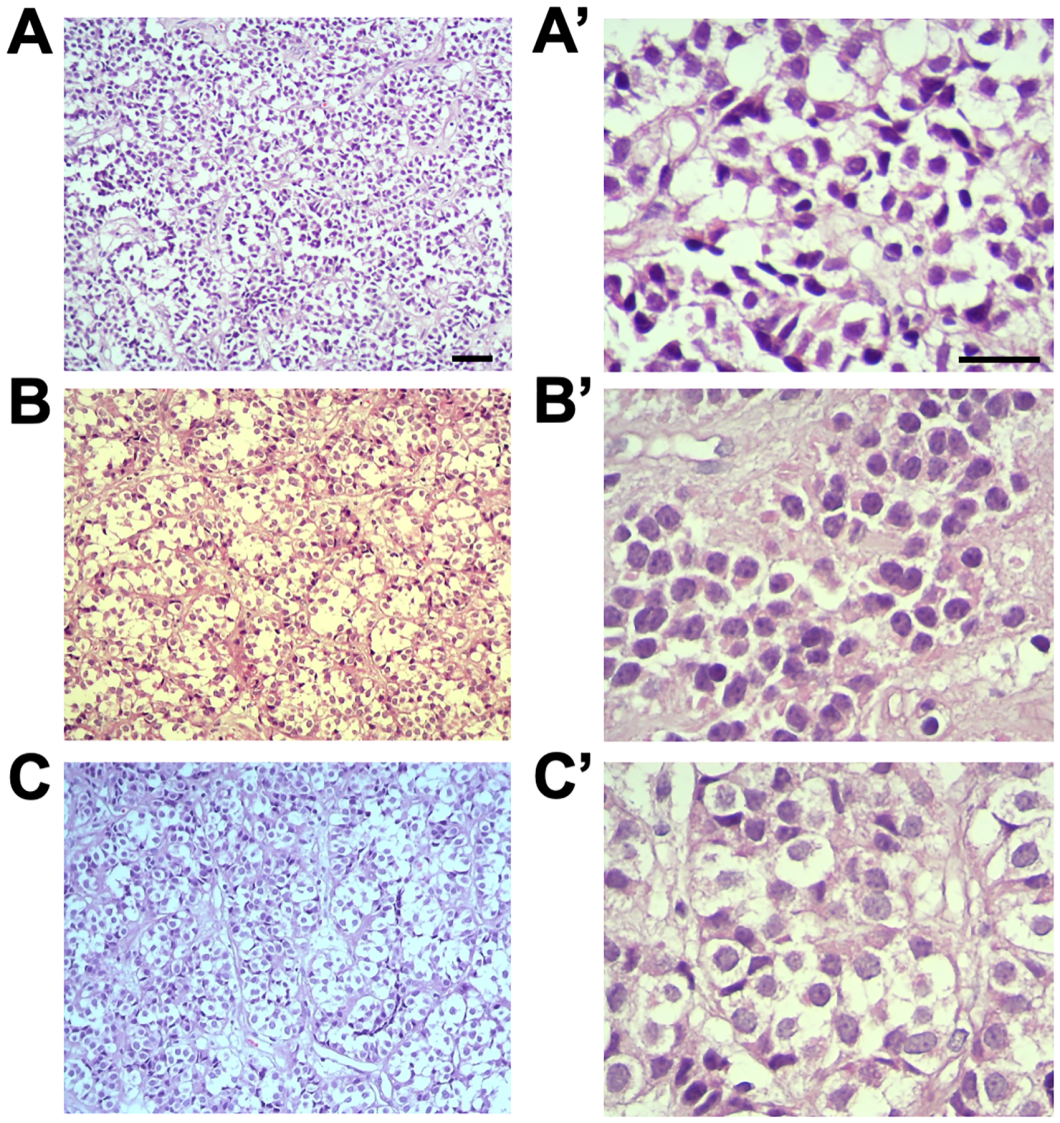
Anterior pituitary adenomas in suicide cases. Histopathological examination was conducted on hematoxylin-eosin stained sections. Representative photomicrographs from three different suicide cases where adenomas were detected. **(A-C)** show panoramic images illustrating the adenomas (scale bar = 100 μm). **A’-C’** present images depicting the cell composition of adenomas (scale bar = 50 μm).

Anterior pituitary weight was higher (t_(20)_ = 2.217, p = 0.046; d = 0.98) in the suicide group (mean mass ± SEM: 216.64 mg ± 16.01) in comparison with the non-suicidal group (mean mass ± SEM: 162.15 mg ± 15.06). Histopathological examination of anterior pituitary sections stained with hematoxylin-eosin revealed the presence of pituitary microadenomas (<10 mm in diameter) (32) in 5 suicide cases (35% of the cases), with none of these tumors found in the non-suicidal cases (**Figure 2**). There was no statistically significant association between the two variables (Fisher’s exact test, two-tailed, p = 0.1154).

### Differences between compartmentalized and free Zn was detected in anterior pituitary of suicide completers

**Figure 3:**
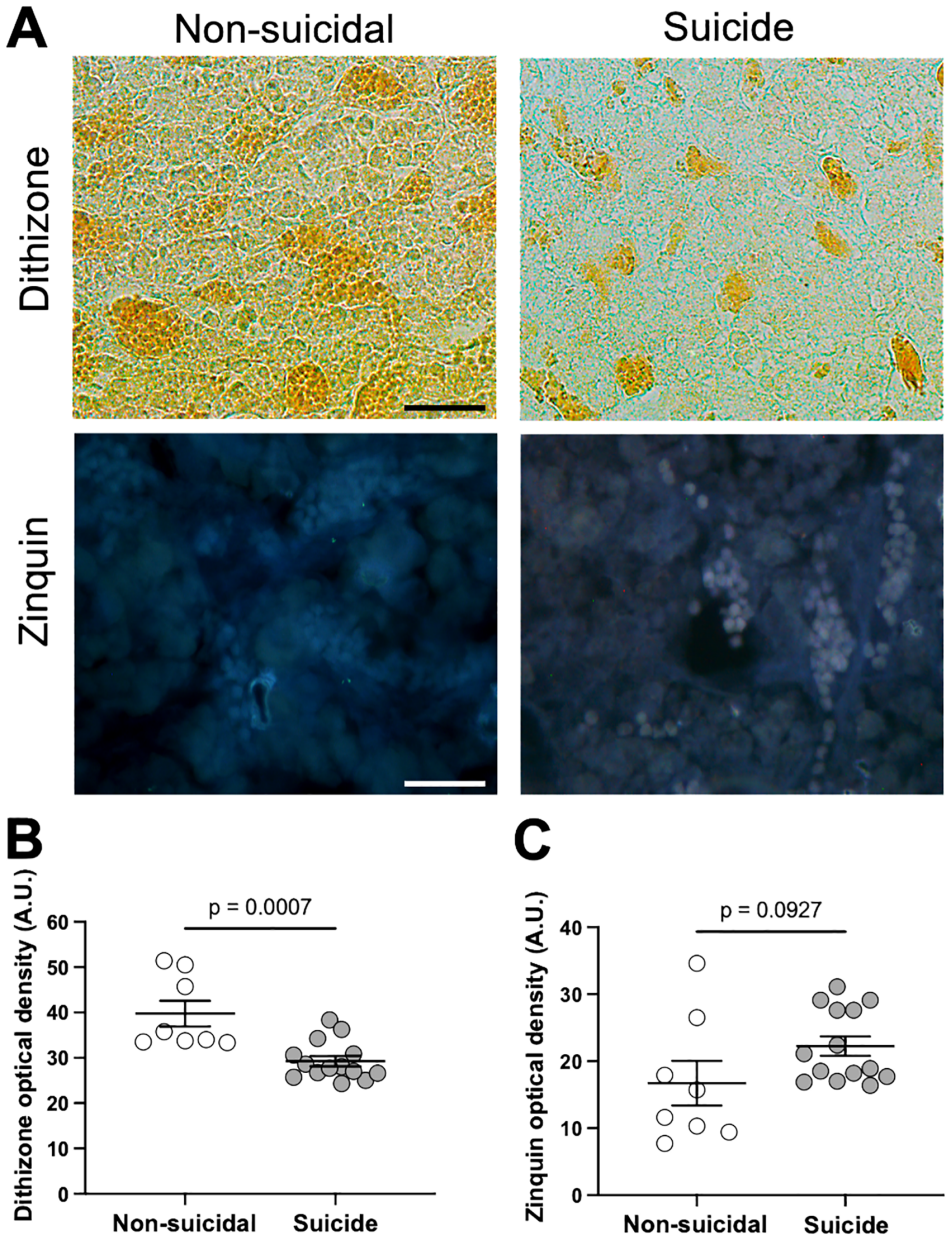
Zn detection in anterior pituitary. **(A)**, Representative photomicrographs of anterior pituitary sections stained with dithizone and zinquin from non-suicidal and suicide cases (Scale bars = 50 μm). **(B)**, Dithizone analysis. Lower optical density for dithizone was detected in suicide cases compared to the non-suicidal group (Cohen’s d test for effect size: d = 1.62). **(C)**, Zinquin analysis. No significant change in zinquin fluorescence was detected between non-suicidal and suicide cases (Cohen’s d test for effect size: d = 0.72). The data were analyzed using a Student’s t-test.

Through dithizone and zinquin staining, we evaluated the Zn homeostasis in the anterior pituitary of suicide completers (**Figure 3A**). Dithizone, a sulfurous organic compound, chelates compartmentalized Zn. Dithizone optical density was lower in suicide cases compared to the non-suicidal group (t_(20)_ = 4.017, p = 0.0007; d = 1.62; **Figure 3B**). However, not all Zn can compartmentalize; it can also exist in a reduced form known as free Zn, which is detected by the lipophilic-sensitive molecule zinquin. Zinquin fluorescence did not change between non-suicidal and suicide cases (t_(20)_ = 1.766, p = 0.0927; d = 0.72; **Figure 3C**).

### Correlation analysis

**Figure 4:**
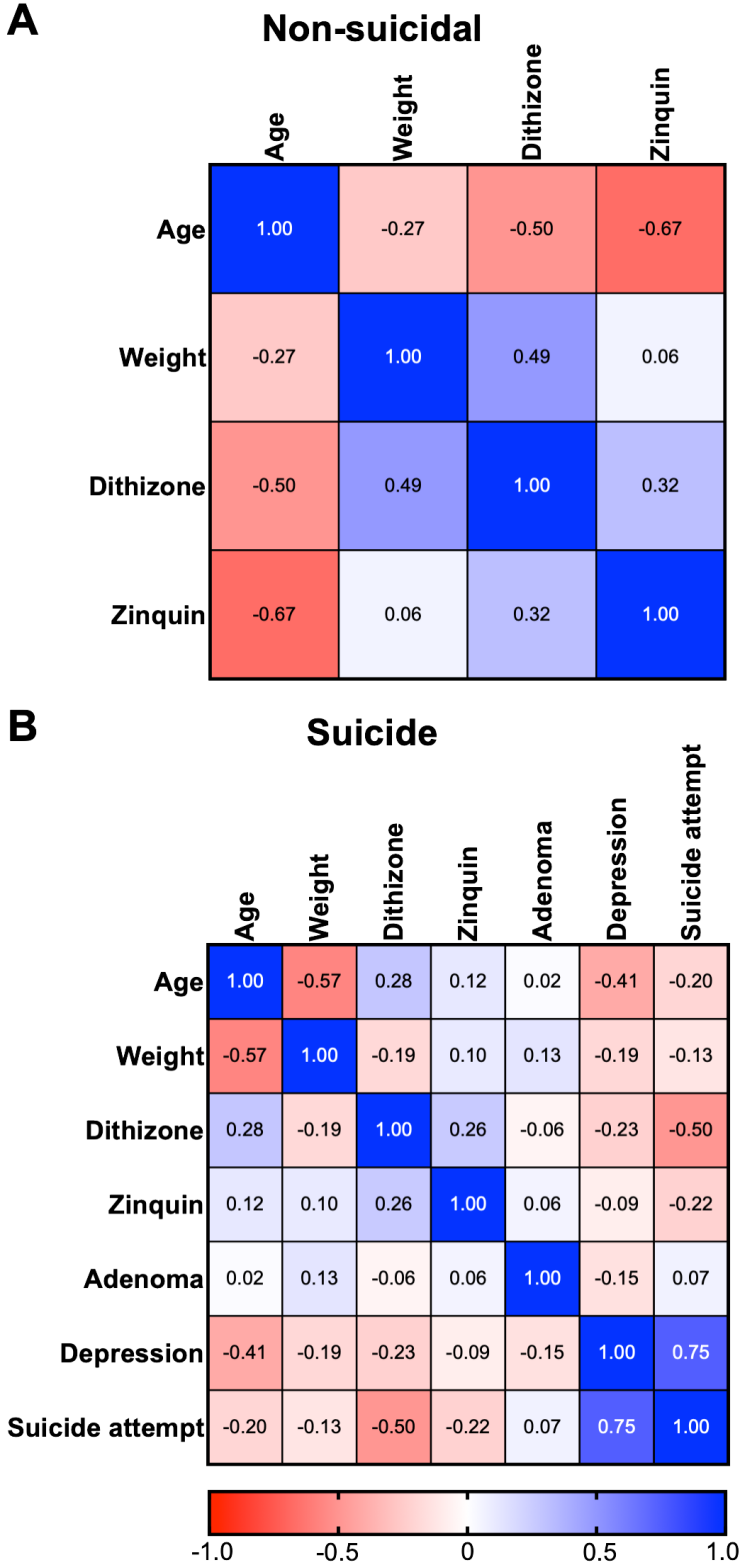
Correlation analysis heat map. **(A)**, Pearson correlations were analyzed for continuous variables (age, pituitary weight, zinquin, and dithizone levels) in non-suicidal cases. A trend was observed indicating a negative correlation between age and zinquin levels (p = 0.068). **(B)**, Spearman correlations were conducted between continuous and categorical variables (depression diagnosis, suicide attempts, and adenoma) in suicide cases. The sex variable was excluded from Spearman correlations due to the lack of female cases. In this group, two significant correlations were identified: a negative correlation between age and weight (p = 0.035), indicating that as age increases in the suicide group, anterior pituitary weight decreases, and a positive correlation between depression etiology and suicide attempts (p = 0.021), suggesting that depression increases the risk of suicide attempts. Each square on the plot illustrates the relationship between two variables—one along the horizontal axis and the other along the vertical axis. Pearson or Spearman coefficients (r) are represented on a color scale ranging from blue to red, corresponding to r = -1 and r = 1, respectively.

Regarding correlation analyses, no significant correlations were detected in the non-suicidal group. However, there was a trend indicating a negative correlation between age and zinquin levels (Pearson r = -0.67, p = 0.068; **Figure 4A**). In the suicide group, two significant correlations were detected. There was a negative correlation between age and weight (Spearman r = -0.57, p = 0.035; **Figure 4B**), suggesting that as age increases in the suicide group, anterior pituitary weight decreases. Also, depression etiology and suicide attempts were positively correlated (Spearman r = 0.75, p = 0.021; **Figure 3**), suggesting that depression increases suicide attempt risk, as previously postulated (1, 38).

In summary, anterior pituitary microadenomas were found in 35% of suicide cases but were absent in non-suicidal cases. Additionally, individuals who died by suicide had lower compartmentalized Zn levels compared to the non-suicidal group (though free Zn levels did not differ). Regarding correlation analyses, the non-suicidal group showed only a trend suggesting a potential negative relationship between age and zinquin levels (free Zn). In contrast, significant correlations were observed in the suicide group: older age was associated with lower anterior pituitary weight, and there was a positive correlation between depression severity and suicide attempts.

## Discussion

In this study, we examined the pituitary glands of 14 suicide completers and 9 non-suicidal cases, finding the presence of adenomas and impaired Zn homeostasis in the anterior pituitary of suicide completers. Pituitary microadenomas were detected in 35% of the suicide cases (5 out of 14) and in none of the non-suicidal cases. Additionally, compartmentalized Zn, but not free Zn, was detected at lower levels in suicide cases compared to the non-suicidal group.

Abnormal stress, particularly when chronic or occurring during specific temporal windows such as childhood (early-life stress), has been identified as a risk factor for suicide (1, 39, 40). Additionally, pituitary dysfunction and abnormal stress have been widely documented, as the HPA axis is a critical neuroendocrine stress regulator. Chronic stress leads to dysfunction at all the components of the HPA axis (41). Beyond biochemical alterations (increased CRH, ACTH, and cortisol levels), abnormal stress also causes anatomical changes in HPA axis components, such as adrenal hypertrophy (42–44) and increased pituitary gland volume (45) observed in both rodents and humans. Interestingly, our research group previously reported mass increases accompanied by hyperplasia in the anterior pituitary gland of suicide completers (18).

In the current cohort, pituitary adenomas were detected in 5 out of 14 suicide cases (35%) and in none of the non-suicidal cases. Most pituitary adenomas (<95%) are sporadic, without a known hereditary origin, and are asymptomatic (46). A study performed in an Ecuadorian population revealed that pituitary adenomas were present in 23% of the population, being more frequent in the age range of 20 to 39 years old (47). These results are similar to those reported in this research, as the mean age of both groups is around 30 years old. Despite the microadenomas prevalence in the suicide group being 35%, when considering the entire studied population, the prevalence is 21.7% (5 out of 23 total cases). This is consistent with reports in the Mexican population, where the prevalence of pituitary adenomas is 15 to 23% of the general population, depending on the diagnostic tool employed (48). Additionally, in the Mexican population, there are increased hyperprolactinemia rates secondary to psychotropic drug treatments in patients diagnosed with psychiatric diseases (49). This may suggest a role of these drugs in the prevalence of pituitary microadenomas in suicide cases. However, the pharmacological information on psychotropic drug exposure was not available for the cases in the present study. Nonetheless, this certainly opens an interesting working hypothesis for further research.

Despite these tumors being relatively prevalent worldwide, approximately 1 case per 1000 of the general population (50), in the Polish population, the presence of pituitary microadenomas has been postulated as a risk factor for suicide (31). In this same study, the adenomas were biochemically characterized, finding that most of them were GH-secreting. Unfortunately, we were unable to perform a similar characterization in the present study.

Among the various consequences that microadenomas could generate, we were interested in their impact on Zn dynamics, since it is known that tumor cells impairs this process (33, 34). Interestingly, Zn levels specifically influence anterior pituitary growth hormone (GH) synthesis and consequently the insulin-like growth factor-I (IGF-I) pathway. Zn deficiency has been associated with both increased and decreased levels of GH in the bloodstream but consistently results in lower circulating concentrations of IGF-I (51). Thus, IGF-I has been postulated as a biomarker for major depressive disorder, as peripheral IGF-I levels might predict future depressive episodes and reflect cognitive dysfunction (52). Interestingly, the GH-containing secretory granules of anterior pituitary cells have been found to possess a high Zn concentration in the rat (10, 53).

In this study, we were unable to biochemically characterize the pituitary microadenomas, which represents a limitation due to the known sex- and age-related differences in this condition. Endocrine-inactive and GH-releasing adenomas were more prevalent in males, whereas prolactinomas, ACTH-releasing adenomas, and TSH-releasing adenomas were more common in females. Except for endocrine-inactive adenomas, all types exhibited a more balanced sex distribution in older age groups. Endocrine-inactive adenomas were most frequent in middle-aged and elderly adults, while prolactinomas peaked in adolescents, young adults, and middle-aged adults. Throughout adulthood, GH-releasing, ACTH-releasing, and TSH-releasing adenomas showed a more uniform distribution (54). These findings highlight another limitation of the study: the lack of female cases, which will be addressed later.

However, beyond Zn’s potential endocrine role in suicide, this element is involved in multiple brain processes, which have been associated as risk factors for suicide (1), including the modulation neurotransmitter function, and can act as a neuromodulator, specifically for glutamatergic transmission, promoting synaptic plasticity (55). This is interesting, since increased protein levels of ZnT isoforms 1 and 4-6 have been reported in the prefrontal cortex of suicide completers (28). These isoforms act as Zn^2+^/H^+^ exchangers in the plasma membrane and membranous organelles, essential for Zn mobilization across these cell structures (11), favoring two phenomena: the vesiculation of Zn and its mobilization into the extracellular space (53). Although we were unable to study the expression of ZnT, further research should investigate it, as it would provide mechanistic explanations for the impaired Zn homeostasis, specifically regarding compartmentalized/free imbalance in the anterior pituitary in suicide reported in this study.

Interestingly, low serum Zn levels have been detected in mental illnesses (56) and in suicide (15). The research conducted by Huang and colleagues (15) reported lower serum Zn levels in individuals with suicidal thoughts in a US study with over 4500 participants, suggesting that lower Zn levels may increase susceptibility to suicidal ideation. This relationship between Zn and suicidal ideation is crucial. In patients diagnosed with major depression, it has been reported that Zn supplementation, in conjunction with common antidepressant treatments, enhances patients’ symptoms (16, 57). Therefore, we hypothesize that studying the neuroendocrine mechanisms of Zn could contribute to a better understanding of the pathophysiology of suicide.

Zn also serves as an anti-inflammatory and antioxidant mechanism in the system (58). In a previous study, we detected impaired antioxidant systems, as evidenced by increased superoxide dismutase activity and reduced nitrite levels (a stable form of nitric oxide, a free radical), without any changes in Zn concentration (18). It has been hypothesized that suicide (1), along with some psychiatric conditions closely related to it, including chronic stress (59), major depression (60), and schizophrenia (61), share a common pathophysiological mechanism: a sustained systemic pro-inflammatory state. Depression and other psychiatric conditions are considered the main etiological causes of suicide in the Mexico City population (40, 62). For Zn to exert its anti-inflammatory or antioxidant effects, it must be free in the cytosol. Following the aforementioned pro-inflammatory hypothesis for suicide, this may explain the reduction in compartmentalized Zn detected in this study, as a compensatory mechanism attempting to reduce inflammatory response.

As mentioned before, the lack of female cases included in this study is a limitation, not only relevant for pituitary microadenomas (as previously discussed) but also for Zn levels. Serum Zn levels are higher in males than in females, both in young and elderly age groups, with significant differences between young males and females (63). Therefore, the findings of this research might not have physiological relevance for understanding the explored histopathological and Zn dynamics mechanisms in females, and further studies must address this issue.

Throughout the manuscript, we have highlighted the study limitations, which are summarized as follows: The main one is the relatively small sample size, which may limit the conclusions drawn. To contextualize the results appropriately, however, we have reported effect sizes (d values). Regarding the adenomas, a primary limitation was the absence of biochemical characterization. This understanding is crucial for delineating the neuroendocrine role of these structures, especially in light of a previous report indicating increased prevalence of microadenomas positive for GH in suicide cases (31), as well as the sex- and age-related differences documented (54). Concerning the Zn studies, the main limitation was the inability to evaluate the levels of ZnTs and ZIPs in the anterior pituitary. This information is essential for comprehending Zn homoestasis and may facilitate the identification of specific mechanisms related to Zn mobilization in suicide, as reported in some brain regions (28). Another limitation is the lack of females in the study (2 non-suicide and 1 suicidal), this is crucial since sex-related differences in Zn levels have been reported in physiological (63) and psychiatric conditions (64, 65). Despite these limitations, the study has notable strengths. Most notably, the study delves into a significant yet under-explored field, examining connections between pituitary microadenomas, suicide, and Zn homeostasis, potentially revealing molecular mechanisms underlying suicidal behaviors. Furthermore, the meticulous use of cost-effective histological and histochemical methods to assess Zn balance and structural integrity in the anterior pituitary enhances data reproducibility, facilitating the generation of valuable datasets for future studies.

In conclusion, our study suggests an association between suicide and the presence of anterior pituitary microadenomas, alongside disrupted Zn homeostasis. Microadenomas in the anterior pituitary have been proposed as a suicide risk factor, supported by our findings. However, further studies are needed to biochemically characterize these structures to better understand their role in known or unknown neuroendocrine mechanisms related to suicidal behavior. Beyond its neuroendocrine implications in disrupting the HPA axis, microadenomas may also affect pituitary Zn dynamics. Zn has emerged as a potential biomarker and therapeutic target for psychiatric conditions, including suicide, providing insights into its neurobiological role. However, little has been studied about Zn, the pituitary, and suicide, with this being one of the first works to do so, thus establishing the relevance of the present research in the field. Nevertheless, additional research should investigate specific mechanisms of Zn dynamics impairment involving ZIPs and ZnTs to better comprehend this phenomenon in suicide. These findings deepen our understanding of the complex pathophysiological mechanisms involved in suicidal behavior, particularly concerning neuroendocrine and inflammatory pathways. Moreover, they underscore the importance of studying diverse populations to identify suicide risk factors associated with varied genetic, biochemical, sociodemographic, and economic profiles.

## Data Availability

The raw data supporting the conclusions of this article will be made available by the authors, without undue reservation.
